# Association between Bullying Victimization and Symptoms of Depression among Adolescents: A Moderated Mediation Analysis

**DOI:** 10.3390/ijerph18063316

**Published:** 2021-03-23

**Authors:** Songli Mei, Yueyang Hu, Mengzi Sun, Junsong Fei, Chuanen Li, Leilei Liang, Yuanchao Hu

**Affiliations:** 1 Department of Child and Adolescent Health, School of Public Health, Jilin University, NO. 1163 Xinmin Street, Changchun 130012, China; meisongli@sina.com; 2 Department of Social Medicine and Health Management, School of Public Health, Jilin University, NO. 1163 Xinmin Street, Changchun 130012, China; 18844194244@163.com (Y.H.); kurosakidomo@163.com (J.F.); 18364166569@163.com (C.L.); liangleileill@163.com (L.L.); 3 Department of Epidemiology and Statistics, School of Public Health, Jilin University, NO. 1163 Xinmin Street, Changchun 130012, China; smz20@mails.jlu.edu.cn

**Keywords:** bullying victimization, social anxiety, depressive symptoms, sleep duration, moderated mediation analysis

## Abstract

Background: Bullying victimization and its effect on symptoms of depression have received attention from researchers, but few studies have considered the potential mechanism. The aim of this study was to examine a moderated mediation model for the association between bullying victimization and depressive symptoms in terms of it being mediated by social anxiety, and investigated whether sleep duration would show moderating effects in this relationship. Methods: In this study, there were 2956 students, who completed three questionnaires, including a bullying victimization scale, as well as a social anxiety and epidemiologic studies depression scale. Results: Bullying victimization’s effects on depressive symptoms were mediated by social anxiety. Furthermore, sleep duration moderated the relationship between bullying victimization and depressive symptoms. Conclusions: The research contributes by clarifying the mechanisms underlying the relationship between bullying victimization and depressive symptoms.

## 1. Introduction

Similar to in most Western countries, mental health problems including depressive symptoms among Chinese adolescents have been identified as important public health issues [1]. In China, 4.41% to 55.7% of teenagers have been classified as suffering from depressive symptoms [2]. Due to the many factors involved, it is difficult to predict which will develop depressive symptoms, although the etiology of depressive symptoms has increased in the last decade [3]. Stressful life events are an accepted predictor of depressive symptoms [4]. Bullying victimization is a typically negative life event.

Bullying victimization is defined as reduplicative passive behavior by one or more individuals over time, and intentionality between (a) perpetrator(s) and a victim [5]. Negative actions, unprovoked rejection and social isolation, attacks, malicious rumors, humiliation and ridicule, and name-calling, can lead to severe distress in the victims. At the same time, the victims cannot defend themselves [6]. Since approximately 32% of school children in 38 regions have reported experiencing emotive persecution and companion victimization, bullying victimization has become a serious public health problem [7]. The detection rate of bullying victims in China is between 2% and 66% [8]. Studies have shown that there are gender differences associated with victimization [9], that older age is a protective factor for victimization experiences [10], and that siblings may represent a negative factor associated with victimization [11]. Among the currently various types of bullying, the most common setting where it occurs is in school [12]. Young people who have been targets of bullying have reported higher standards of depressive symptoms compared to non-victimized peers. Furthermore, the seriousness of depression in youth victims has been shown to be associated with the degree and severity of victimization [13].

According to a biological theory of depression, a stressful life and bullying could induce a series of psychological and physiological changes. Some studies have shown that victims are prone to mental health problems such as anxiety and depression [14,15]. Many longitudinal studies have reported these associations [16,17] and have indicated that there is a longitudinal relationship. There also seems to be a dose–response relationship, with children who have been bullied for a long time tending to have worse mental health outcomes [18].

Bullying victimization can affect depressive symptoms in victims directly, as well as indirectly through mediating variables such as social anxiety. Social anxiety is recognized as a crucial factor for understanding interpersonal behavior [19], as it refers to the tension in social situations [20]. Studies have reported that teens with social anxiety are more likely to suffer from comorbid psychologic problems, impaired emotional development, and unhealthy relationships [21]. Social anxiety usually strikes in early teens (10–13 years), and therefore, puberty is a critical period for the development of social anxiety, because a number of changes including physical changes, sociocognitive maturation, the school environment, and the increasing importance of social interactions with peers could contribute to the emergence of social anxiety, especially during the teenage years [22].

Victims of bullying have been identified as individuals who experience more psychological symptoms [23]. Bullying victimization can increase social anxiety, and, consequently, young people feel alienated and isolated from social support, which increases social anxiety levels [24]. According to cognitive behavioral models, social anxiety is affected by their fear of negative estimation in potentially social-evaluative situations. A longitudinal study suggested that bullying victimization could forecast subsequent social anxiety [25]. Similarly, Storch et al. found that teenagers between 13 and 16 years old who had experienced overt or relationship victimization, or high levels of both had higher social anxiety levels than those adolescents who had suffered only obvious or no victimization [26]. On most occasions, social anxiety usually precedes depressive symptoms [27]; a longitudinal research reported that depressive symptoms were predicted by social anxiety [28]. Other studies have shown that social anxiety is an influencing factor for depressive symptoms and predicting the occurrence of depressive symptoms in individuals [29]. Social anxiety can damage an individual’s ruminative thinking, meta-evaluation of emotions, or self-efficacy, and induce depressive symptoms [30]. According to the interpersonal theory of depression among youth, many interpersonal problems are influencing factors for depressive symptoms [31]. Rubin et al.’s transactional model suggested that social anxiety and depression were the result of social withdrawal, which was similar to the findings of the above-mentioned studies [32]. Therefore, based on these findings, we proposed Hypotheses 1, i.e., social anxiety can mediate the association between bullying victimization and depressive symptoms.

Junior high school, which is considered to be one of the most important physical and social environments, has a significant impact on the socialization of teenagers [33]. Due to the intense academic pressure that exists in China, students tend to sometimes sacrifice sleep time in order to study longer. Recent studies have shown that sleep time is essential for regulating emotions [34]. Sleep time is one of the criteria for measuring sleep quality and an influencing factor for mental health [35]. According to the theoretical model of self-control resources, it is believed that individual self-control depends on self-control resources [36]. Students who experienced negative life events such as bullying victimization over a long period of time could be in a state of self-depletion. Self-depletion refers to “a state in which one does not have all the resources normally possessed” [37]. Sleep can restore and supplement self-control resources [38]. After bullying victimization has consumed individual self-control resources, an insufficient sleep duration cannot supplement the resources in time. Sampasa-Kanyinga, H., found that higher levels of bullying involvement could lead to short sleep duration [39]. Another study found that bullied adolescents reported shorter sleep durations and a higher incidence rate of insomnia [40]. Thus, as the degree of self-depletion increases, the possibility of causing depressive symptoms significantly increases.

**Figure 1:**
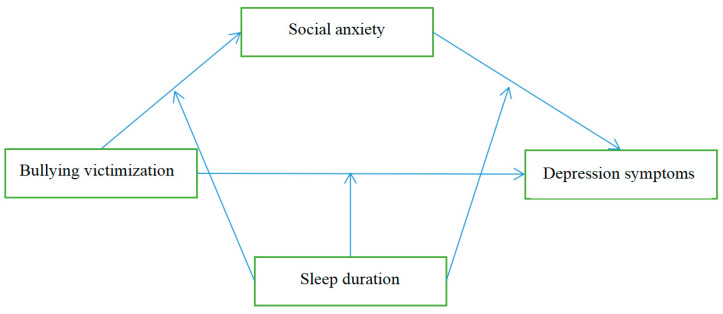
Logical frame diagram.

Furthermore, a recent study suggested that sleep duration played a crucial role in regulating mood [34]. Without adequate healthy sleep, negative emotional responses are significantly enhanced, while positive responses to positive events tend to be diminished [41]. The results showed that sleep deprivation exacerbated passive emotions and reduced positive emotions after achieving a target event in another recent sleep deprivation study [42]. Thus, social anxiety’s role in mediating the relationship between the bullying of victims and depressive symptoms may vary depending on sleep duration. Empirical studies have shown that sleep duration can moderate the effect of social anxiety on depressive symptoms [43]. From a behavioral perspective, sleep deprivation may also reduce adolescents’ goal-oriented behaviors and motivations, which may further exacerbate social withdrawal [44], thereby enhancing the effect of social anxiety on depressive symptoms. As a result, we proposed the following two hypotheses. Hypotheses 2: sleep duration can moderate the indirect association between bullying victimization and depressive symptoms via social anxiety. Hypothesis 3: sleep duration has an influence on the direct effect of victimization on depressive symptoms (Figure 1).

## 2. Methods

### 2.1. Study Participants

A total of 2956 middle school students, aged 10 to 15 years old, were recruited for this cross-sectional study from December 2017 to January 2018. In Stage 1, with a stratified-random sampling method, three cities were chosen from Province Jilin and were categorized into developed, developing, and undeveloped cities based on the GDP status of each city. In Stage 2, each city was further divided into suburban and urban areas based on their geographical locations, which made a total of six unique areas, and then, one district was randomly selected from these unique areas. In Stage 3, two junior high schools were randomly selected from each urban area, and two junior high schools were randomly selected from each suburban area. In Stage 4, we randomly selected two classes from each grade (grades 7 to 9) in each school. At least 80 students were selected from each grade. Thus, the number of participants was 80 × 3 × (2 + 2) × 3 = 2880. We calculated the sample size using a non-response rate of 10%; therefore, we added 8–10 students in each grade. A total of 3168 students were invited to participate in the present study, and 2956 of them completed all the questionnaires, representing a response rate of 93.3%. The non-responses were mainly caused by students’ absence during data collection.

To ensure the quality of the study, all the schools that had been chosen communicated with the parents of the selected students to ensure that they were available for the entire study.

### 2.2. Measures

#### 2.2.1. Social Anxiety Scale

In this study, the first questionnaire used was the Social Anxiety Subscale of the Self-Consciousness Scale compiled by Scheier Fenigstein and Buss in 1975 [45]. Chinese research showed that the Self-Consciousness Scale had good measurement indicators [46]. The Social Anxiety Subscale was comprised of six situational questions to examine the level of social anxiety of the participants. Each question was answered on a 4-point Likert scale, where 0 = not like me at all, 1 = like me a little, 2 = like me somewhat, and 3 = like me a lot. In the end, the scores of each question were added together to obtain the final result, with a higher score indicating more severe social anxiety. The Cronbach’s α coefficient was 0.84.

#### 2.2.2. Epidemiologic Studies Depression Scale

The Center for Epidemiologic Studies Depression Scale (CES-D) is a 20-item indicator [47]. The Chinese version was developed by four Chinese researchers who were proficient in both English and Chinese. Ever since then, it has been a popular instrument that Chinese researchers use in relevant research fields with both high validity and reliability. The response options for each item range from 0 to 3 (0 = little or no time, 1 = some or little of the time, 2 = medium or most time, and 3 = most or almost all the time). The score ranges from 0 to 60, with high scores indicating increased depressive symptoms [48]. The Cronbach’s α coefficient was 0.87.

#### 2.2.3. Bullying Victimization

We used the Chinese translated version of a questionnaire that measures bullying victimization, which was developed by Solberg in a previous study [49]. The instrument referred to victimization behavior in the last two months. The version of the questionnaire has been proven to have high validity, and it is widely used among Chinese adolescents [50]. The response options were scored on a 3-point Likert scale, ranging from 1 (never) to 3 (always), with higher scores on the bullying victimization indicating more elevated or higher levels of victimization. The questionnaire includes six questions: “being teased”, “being asked for belongings”, “deliberately excluded or isolated from group activities”, “being threatened and intimidated”, “being beaten, kicked, pushed, squeezed or locked in the house”, and “being teased for physical defects or looks”. A higher total score indicates that the participant has experienced more severe bullying in the past two months. The Cronbach’s α coefficient was 0.89.

#### 2.2.4. Sleep Duration

In order to measure the sleep duration in the past month, the participants were asked the following question: “How many hours do you sleep every night on average in the past month?”.

### 2.3. Statistical Analyses

The statistical analyses were performed using SPSS 24.0. A Pearson correlation analysis was used to test the relationship between the study variables. The mediating effects were tested by using PROCESS of SPSS Macro [51]. The bootstrap method was used to re-sample 5000 samples, and 95% confidence intervals (CI) were calculated. All the statistical tests were conducted by two-tailed tests, and a *p* value less than 0.05 was considered to be statistically significant.

### 2.4. Ethics Statement

This study was approved by the Institutional Review Board of the School of Public Health, Jilin University (2017-09-06).

## 3. Results

### 3.1. Sample Characteristics

The sample included 1494 males (50.5%) and 1462 females (49.5%). The age range of the subjects was 10–15 years, with an average age of 13.39 ± 1.03 years. There was a significant difference between the proportion of only-child students (n = 1966, 66.5%) and the proportion of non-only-child students (n = 990, 33.5%). In terms of residence, the study included 1459 urban participants (49.4%) and 1497 rural participants (50.6%) (see Table 1).

### 3.2. Descriptive Analyses

There was a positive correlation between bullying victimization and social anxiety in our study (r  =  0.121, *p*  <  0.01), and symptoms of depression (r  =  0.491, *p*  <  0.01). Sleep duration was negatively associated with social anxiety (r  =  −0.081, *p*  <  0.01) and symptoms of depression (r  = −0.460, *p*  <  0.01) (see Table 2).

### 3.3. Testing for the Mediation Effect

The Model 4 test in PROCESS (Hayes and Preacher) was used to examine whether social anxiety mediated the relationship between bullying victimization and depressive symptoms. In Model 1, bullying victimization was significantly associated with depressive symptoms (*β* = 0.11, *p* < 0.01). In Model 2, bullying victimization was significantly associated with social anxiety (*β* = 0.44, *p* < 0.01). In Model 3, when we controlled for bullying victimization, social anxiety was significantly associated with depressive symptoms (*β* = 0.46, *p* < 0.01). (see Table 3).

### 3.4. Testing for the Moderated Mediation Effect

**Figure 2:**
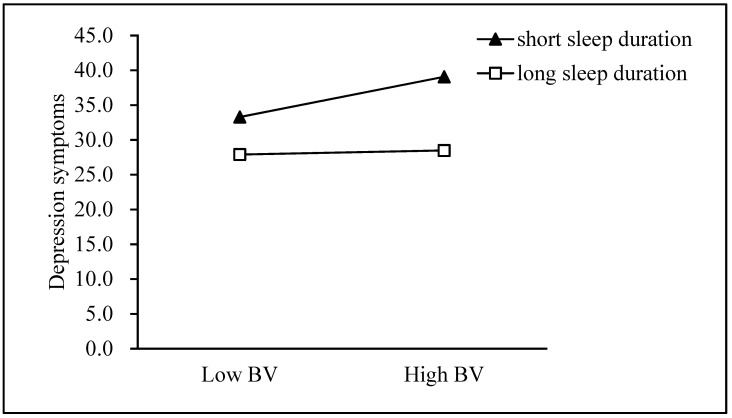
Sleep duration moderates the effect of bullying victimization on depressive symptoms. BV = Bullying victimization

**Figure 3:**
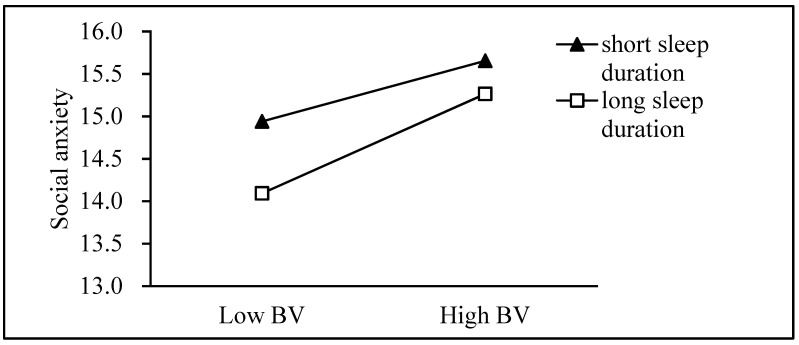
Sleep duration moderates the effect of bullying victimization on social anxiety. BV = Bullying victimization.

**Figure 4:**
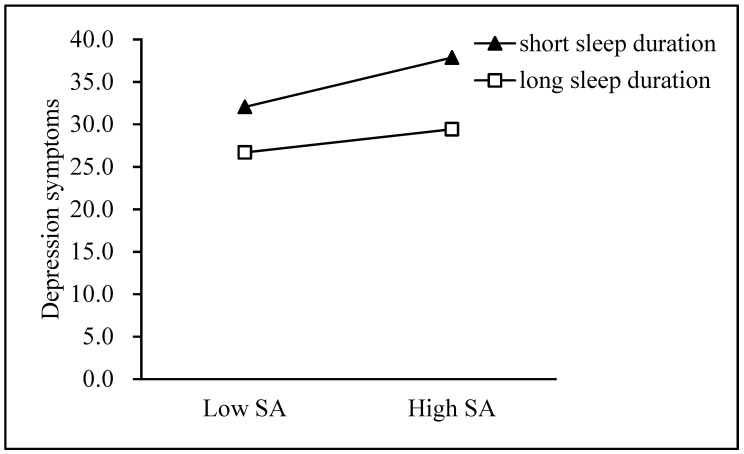
Sleep duration moderates the effect of social anxiety on depressive symptoms. SA = Social anxiety.

First, the Model 59 test in the application process of this study tested the theoretical hypothesis model (see Table 4), and Model 1 was significant (F = 734.169, *p* < 0.001, R^2^ = 0.427). Bullying victimization was positively correlated with depressive symptoms, and sleep duration was negatively correlated with depressive symptoms. We used a simple slope analysis to prove the significant interaction between sleep time, one standard deviation below the mean and one standard deviation above the mean (see Figure 2). Model 2 was significant (F = 190.494, *p* < 0.001, R^2^ = 0.141). The interaction item between the bullying of the victim and sleep time was correlated with social anxiety, indicating that sleep time regulated the relationship between the bullying of the victim and social anxiety (see Figure 3). Finally, Model 3 was significant (F =538.474, *p*< 0.001, *R*^2^ =0.477). The interaction term between social anxiety and sleep duration was related to symptoms of depression (see Figure 4).

As shown in Figure 2, for individuals with short sleep duration, higher bullying victimization was associated with higher depressive symptoms (*β*_short_ = 0.422, *t* = 29.419, *p* < 0.001, *β*_long_ = 0.060, *t* = 2.403, *p* = 0.016).

Bullying victimization predicted social anxiety for youth with longer sleep duration according to the test of simple slopes (see Figure 3) (*β*_short_ = 0.374, *t* = 5.367, *p* < 0.001, *β*_long_ = 0.613, *t* = 5.070, *p* < 0.001).

Social anxiety was associated with depressive symptoms, and sleep duration moderated this relation (Figure 4) (*β*_short_ = 0.357, *t* = 17.493, *p* < 0.001, *β*_long_ = 0.100, *t* = 4.561, *p* < 0.001).

## 4. Discussion

Junior high school students are in a pivotal transition period; individuals experience many physical and psychological changes during puberty [52]. As a result, the negative effects on junior high school students due to bullying were more serious compared to those for other demographic groups [10]. We examined the mediating effect of social anxiety on the relationship between depressive symptoms and bullying victimization, as well as the moderating role that sleep duration played in the relationship. Our findings are conducive to a better understanding of the underlying mechanisms of depressive symptoms, which could help to improve mental health among junior high school students and to prevent the development of their depressive symptoms [53].

As expected, Hypothesis 1 was confirmed; i.e., the effect of bullying victimization on depressive symptoms was mediated by social anxiety. The simple mediation analysis showed that teens who were regularly bullied were likely to develop social anxiety [54], which could lead to the development of depressive symptoms [55]. One explanation for this is that socially awkward adolescents have trouble socializing with others and end up alienated form their peers, which can strip away their confidence, downsize their self-esteem, induce feeling of hopelessness, and, consequently, result in social anxiety and depression [56]. Socially anxious adolescents often deliberately avoid social interactions and isolate themselves in social seclusion and lonely environments [57], which can aggravate their depressive symptoms [58]. Besides, trajectories of social anxiety have been discovered to be a predictor of the severity of depressive symptoms in people [59]. Consistent with existing theories and past studies on the effects of social isolation, it is hard for people with social anxiety to think about unpleasant past experiences, and they become further upset [60].

Our study showed that sleep duration played a moderating role, supporting Hypotheses 2 and 3. Specifically, the shorter the sleep duration, the higher the likelihood for someone developing depressive symptoms. The results suggest that adolescents who sleep longer may be protected to some extent from the negative effect of bullying or social anxiety on depressive symptoms. These findings are consistent with the “sleep to forget and sleep to remember” (SFSR) hypothesis [43]. It indicates that optimal sleep can reduce the mood impact of stressful events, for example, bullying victimization [61]. Other studies have reported that short sleep duration amplified the negative influences of bullying on depressive symptoms, while long sleep duration mitigated the negative effects [62]. In a study by Tu et al., longer objective sleep duration were found to protect adolescents from internalized symptoms of low but less severe peer victimization [63]. A possible explanation based on the above results is that the feeling and processing of emotions would be affected by a short sleep duration [64]. The normal regulation of the limbic system fails after sleep deprivation, leading to enhanced responsiveness to negative information. As prefrontal activation decreases, emotion regulation becomes dysfunctional. Healthful sleep restores the integrity of the amygdala connections in the medial prefrontal cortex, which is crucial in emotional regulation, as well as brain activity and adaptive processing [65]. However, our results showed that the longer the sleep duration, the higher the likelihood for someone developing social anxiety. It was surprising to find that the effect in Figure 3 contrasted with that in Figure 2 and Figure 4. Perhaps, neither long nor short sleep duration can be considered to be high-quality sleep and are all part of sleep disturbances [66]. Emerging research suggests that certain mental processes can lead to the link between anxiety and sleep [67]. Anxiety sensitivity (AS) is defined as the apprehensive physical sensations associated with anxiety [68]. For instance, if someone feels their body is hurt (high AS), they will respond with elevated levels of anxiety, resulting in the development of anxiety disorders such as social anxiety. AS is related to a lack of sleep, including poor sleep quality [69] and longer sleep delays [70], possibly because participants recorded sleep latency as average sleep duration.

Our study has several limitations. First, our study does not provide evidence for causality due to its cross-sectional design. Second, all the data used in this study were the result of self-reported questionnaires; therefore, there are questions of subjectivity, validity, and reliability. Finally, social anxiety had a partial mediating effect on the relationship between bullying and depressive symptoms, indicating that there were other mediating variables. In addition, in this study, only the moderating effect of sleep duration was determined. In the future, other relevant factors could be considered. For example, Internet addiction has been identified as an important public health issue and has been linked to depressive symptoms, bullying, and social anxiety.

## 5. Conclusions

The current research investigated the complex relationship between bullying victimization and social anxiety for forecasting depressive symptoms in a sample of junior high school students. The findings support the theory that there is a conditional indirect relationship between bullying victimization and depressive symptoms, with a moderated mediation effect of sleep duration through social anxiety, suggesting the need for psychological interventions for adolescents with bullying victimization and low sleep quality.

## Figures and Tables

**Table 1:** Demographics of the study population.

Characteristic	n	%
Age	13.39 ± 1.03	-
Gender		
male	1494	50.5
female	1462	49.5
Only child		
yes	1966	66.5
no	990	33.5
Residence		
urban	1459	49.4
rural	1497	50.6
Grade		
one	987	33.4
two	983	33.3
three	986	33.3
Residence in school		
yes	421	14.2
no	2535	85.8

**Table 2:** Descriptive statistics and correlations among variables.

Variables	*M*	*SD*	1	2	3	4
Bullying victimization	6.55	1.36	1			
Social anxiety	14.75	5.04	0.121 **	1		
Depression	31.99	10.42	0.491 **	0.276 **	1	
Sleep duration	7.80	1.32	−0.232 **	−0.081 **	−0.460 **	1

** *p* < 0.01. Adjusted for gender, residence, age, and only-child status.

**Table 3:** Mediation analysis of bullying victimization’s effect on depressive symptoms.

Independent Variables	Model 1 (Depressive Symptoms)		Model 2 (Social Anxiety)		Model 3 (Depressive Symptoms)	
	*β*	*t*	*β*	*t*	*β*	*t*
Bullying victimization	0.11	5.81 **	0.44	6.53 **		
Social anxiety					0.46	14.18 **
*R* ^2^	0.110		0.143		0.299	
*F*	33.73 **		41.252 **		251.027 **	
*Model effect*	Effect		SE		*t*	
*Total effect*	1.85		0.12		15.23 **	
*Direct effect*	1.62		0.12		14.01 **	
*Indirect effect*	0.23		0.05		95% CI (0.14–0.33)	

** *p* < 0.01. Adjusted for gender, residence, age, and only-child status.

**Table 4:** Testing the moderated mediation effect of bullying victimization on depressive symptoms.

Independent Variables	Model 1 (Depressive Symptoms)			Model 2 (Social Anxiety)			Model 3 (Depressive Symptoms)		
	*β*	*SE*	*t*	*β*	*SE*	*t*	*β*	*SE*	*t*
Bullying victimization	0.24	0.13	14.14 ***	0.13	0.08	5.95 ***	0.22	0.13	13.29 ***
Sleep duration	−0.33	0.12	−22.33 ***	−0.07	0.07	−3.45 ***	−0.32	0.11	−22.40 ***
Bullying victimization * sleep duration	−0.29	0.06	−16.95 ***	0.09	0.04	2.28 **	−0.29	0.06	−17.10 ***
Social anxiety							0.21	0.03	15.20 ***
Social anxiety * sleep duration							−0.08	0.02	−5.84 ***
*R* ^2^	0.427			0.141			0.477		
*F*	734.169 ***			190.494 ***			538.474 ***		

** *p* < 0.01. *** *p* < 0.001. Adjusted for gender, residence, age, and only-child status.

## Data Availability

The data presented in this study are available on request from the corresponding author.
